# Effects of Asymmetric Vibration Frequency on Pulling Illusions

**DOI:** 10.3390/s20247086

**Published:** 2020-12-10

**Authors:** Takeshi Tanabe, Hiroshi Endo, Shuichi Ino

**Affiliations:** 1 Human Informatics and Interaction Research Institute, National Institute of Advanced Industrial Science and Technology (AIST), Central 6, 1-1-1 Higashi, Tsukuba 305-8566, Japan; s-ino@aist.go.jp; 2 Research Promotion Division of Information Technology and Human Factors, National Institute of Advanced Industrial Science and Technology (AIST), Central 1, 1-1-1 Umezono, Tsukuba 305-8560, Japan; hiroshi-endou@aist.go.jp

**Keywords:** Illusory force sensation, asymmetric vibration, voice-coil-type vibrator, nongrounded haptic interface

## Abstract

It is known that humans experience a haptic illusion, such as the sensation of being pulled in a particular direction, when asymmetric vibrations are presented. A pulling illusion has been used to provide a force feedback for a virtual reality (VR) system and a pedestrian navigation system, and the asymmetric vibrations can be implemented in any small non-grounded device. However, the design methodology of asymmetric vibration stimuli to induce the pulling illusion has not been fully demonstrated. Although the frequency of the asymmetric vibration is important, findings on the frequency have not been reported. In this study, we clarified the influences of the effects on the pulling illusion based on the investigation of asymmetric vibration frequency differences. Two psychophysical experiments that related to the frequency of asymmetric vibration were performed. Experiment I showed that the illusion occurs for specific vibration waveforms at 40 Hz and 75 Hz. As a result of Experiment II, the threshold was the lowest when the frequency was 40 Hz, and highest when the frequency was 110 Hz. This result supports the previous hypothesis that the Meissner corpuscles and the Ruffini endings contribute to the illusion, while the Pacinian corpuscles do not.

## 1. Introduction

Haptic sensation is an important sensory organ for detecting mechanical stimuli applied to the body. By artificially presenting information to the haptic sensation, it is possible to induce movements and behaviors. Interfaces that do this are referred to as haptic interfaces. In recent haptic interfaces, methods of generating an illusory force have been proposed [1,2,3,4].

**Figure 1:**
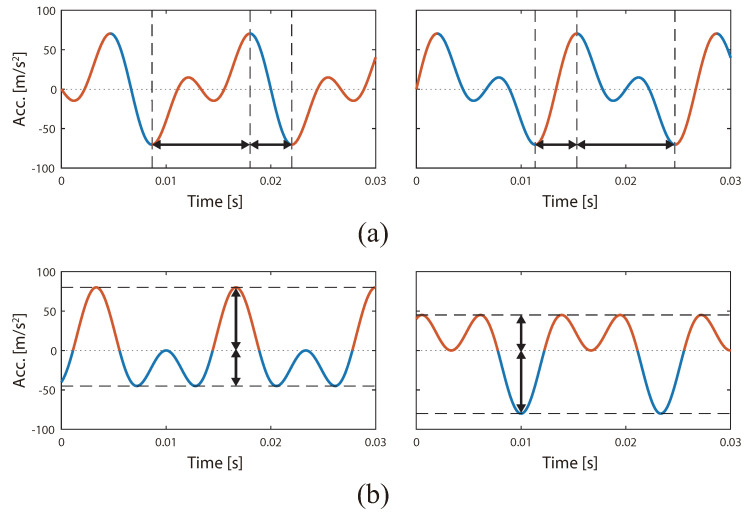
Examples of asymmetric vibration waveforms. (**a**) The waveform is asymmetrical along the time axis because the vibration profiles of the rising and falling accelerations differ with respect to each other. (**b**) The waveform is asymmetric along the amplitude direction because the positive and negative vibration profiles differ with respect to each other.

The sensory properties of humans are nonlinear. Although humans can detect strong stimuli, it is difficult for them to perceive weak stimuli. Amemiya et al. reported that the illusory force was induced by presenting vibrations with asymmetric acceleration [1]. In this paper, vibrations in which the amplitude or change of acceleration differs depending on the polarity are called asymmetric vibrations (Figure 1). When asymmetric vibrations are stimulated, the human perceives a rapidly accelerating asymmetric vibration. By contrast, asymmetric vibrations at low acceleration values are not perceived since low acceleration values cannot be detected. Amemiya et al. demonstrated that users perceive asymmetric vibration stimuli as an illusory pulling force (referred to as a pulling illusion) [1]. The pulling illusion is different from the kinesthetic illusion caused by vibrational stimulation of tendons [5] because the pulling illusion requires stimulation of the fingertips rather than the tendons. The pulling illusion is expected to be used in force display for wearable and mobile devices because it can be induced by a small nongrounded device [6,7,8,9,10]. Owing to this advantage, the pulling illusion has been applied to a pedestrian navigation system for the visually impaired [2,11]. Moreover, it is also applied to the haptic feedback of virtual reality (VR) systems [12,13], and these technologies are also useful for digital twins [14].

However, the design methodology of asymmetric vibration stimuli to induce the pulling illusion has not been fully demonstrated. Moreover, the general effect of the frequency of asymmetric vibration on the illusion has not yet been clarified. In general, it is known that mechanoreceptors have different nerve firing thresholds depending on the frequency of vibration [15]. Thus, the threshold that triggers the illusion may vary depending on the frequency of asymmetric vibration. If we can clarify that the threshold that the illusion occurs depends on this frequency, it would be also possible to clarify the frequency that can efficiently induce the illusion with a smaller amount of energy. This finding helps create a more compact vibrator for generating asymmetric vibrations, and it serves as a design guideline for the frequency band of the vibrator. Therefore, the relationship between the illusion and the frequency must be clarified.

Lately, to induce this illusion, voice-coil type vibrators have been used [6,7,8,9,10] because vibrators are inexpensive and easily available [16]. Although some of these previous studies have reported the effect of frequency [8,9], only the input signal to the vibrator has been controlled between frequency conditions, and the acceleration profiles that output from the vibrator were not controlled. Voice-coil-type vibrators have a resonant frequency because of the spring component [17]; the gain and phase between the input and the output change significantly near the resonant frequency. Therefore, even if the input signal is controlled, the acceleration profile significantly changes between frequencies [6]. Previous studies have not clarified whether the effect of the frequency of asymmetric vibration is due to perceptual characteristics of a human, or the mechanical characteristics of the vibrator.

On the other hand, we confirmed that the asymmetry of the vibration waveform (an acceleration profile per cycle) affects the illusion [18]. Specifically, the illusion occurred when the differences in the rates of change of the acceleration per unit time between the positive and negative directions were maximized, as shown in Figure 1a. Conversely, the illusion did not occur when the differences of the acceleration amplitudes between positive and negative directions were maximized, as shown in Figure 1b. Thus, asymmetry of the vibration waveform, that is, asymmetry in the time axis direction, is more important for inducing the pulling illusion.

At first, when the relationship between the illusion and the frequency is clarified, it is necessary to investigate whether the vibration waveform, in which the illusion occurs, has a dependence on frequency. In our previous study, although the illusion was induced by the vibration whose waveform was asymmetric in the time axis direction, this result has only been confirmed at one frequency condition [18]. If the nonlinear sensory properties of humans contribute to the pulling illusion, the illusion may be induced by presenting the vibration of which the waveform is asymmetric even if the frequency changes. Thus, it is hypothesized that the frequency dependence of the vibration waveform is small. However, before the evaluation of the relationship between the frequency and the illusion, it is necessary to verify the frequency dependence of the vibration waveform at the frequency at which illusion effects occur the least. Therefore, we verified the effect of the vibration waveform at two typical frequencies wherein the occurrence of the illusion was reported (Experiment I). Subsequently, to clarify the effect of the frequency of the asymmetric vibration against the pulling illusion, we investigated the relationship between the frequency and the illusion (Experiment II).

In previous work [18], we reported the development of the experimental device to evaluate the pulling illusion, and the relationship between asymmetry of the vibration waveform (phase difference) and the illusion. In the current paper, we follow on this work by reporting on frequency dependence of vibration waveform, and the effect of frequency against threshold that induced the pulling illusion. The main contributions of this study involve the clarification of the above findings on the frequency of asymmetric vibration in the pulling illusion based on a quantitative evaluation that controls the asymmetric vibration stimuli.

## 2. Experiment I: Effects Owing to Frequency and the Vibration Waveform

Experiment I investigated the effects attributed to the frequency and the vibration waveform. To clarify the basic effect of the frequency and the vibration waveform, a psychophysical experiment was performed with two typical frequencies at which the occurrence of the illusion was reported.

### 2.1. Method

#### 2.1.1. Participants

Sixteen participants, aged 20–55 years (mean: 30.4 years, standard deviation (SD): 12.7 years, four females) participated in the experiment. All participants were right-handed. This experiment was approved by the institutional review board of the National Institute of Advanced Industrial Science and Technology (AIST), and the procedures were performed in accordance with the Declaration of Helsinki. Informed consent was obtained from all individual participants included in the study.

#### 2.1.2. Stimuli

We confirmed that the illusion can be induced by presenting the asymmetric vibrations that consists of two frequency components [18]. The basic model of the asymmetric vibration based on this finding is as follows:

(1) $$\begin{array}{c} \begin{array}{c} {\ddot{x}}_{ref}=A_{1}\sin\left(\omega t\right)+A_{2}\sin\left(2\omega t+\phi_{0}\right)\\ \omega \;=2\pi f \end{array} \end{array}$$

where $\phi_{0}$ is the phase difference between the fundamental wave and the second harmonic, $A_{1}$ and $A_{2}$ are the amplitudes of the accelerations of the two components, ω is the angular frequency, and *f* is the frequency of the fundamental wave (referred to as fundamental frequency). In this study, this basic model was used for asymmetric vibration stimuli because the illusion occurred at minimal levels. More details of the basic model of asymmetric vibration can be found in [18].

**Figure 2:**
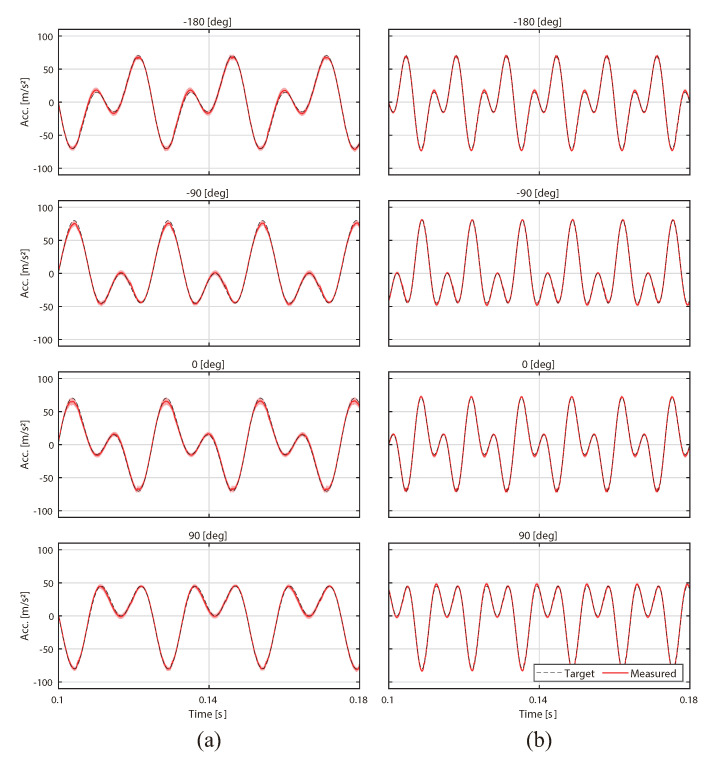
Typical example of an acceleration profile of the fingertips (in Equation (1), $\phi_{0}=$−180, −90, 0, 90 deg, $A_{1}=A_{2}=$ 40 $m/s^{2}$, (**a**) f= 40, (**b**) f= 75 Hz).

According to Equation (1), the waveform changes by changing the phase difference $\phi_{0}$ (Figure 2). The phase difference $\phi_{0}$ can control the asymmetry of the waveform. In our previous study, we clarified that the change that incurs from the instant at which the illusion occurs to the instant at which the illusion does not occur corresponds to fine changes in the phase difference [18]. When the phase differences are −180 deg and 0 deg, the waveform is asymmetrical with respect to the time axis wherein the rising and falling times of acceleration are different (Figure 1a). When the phase differences are −90 deg and 90 deg, the waveform is asymmetric along the amplitude direction because the peak of the amplitude between the positive and negative sides of the axis differ (Figure 1b). When the fundamental frequency is 75 Hz, the illusion occurs at −180 deg and 0 deg, however, the illusion did not occur at −90 deg [18]. In other words, at 75 Hz, asymmetry in the time axis direction is effective in inducing the illusion. To evaluate the effect of frequency and the vibration waveform, Experiment I changed the fundamental frequency *f* and the phase difference $\phi_{0}$ (Figure 2). In addition, it is worth noting that the waveform changed even if the acceleration amplitudes $A_{1}$ and $A_{2}$ changed. If $A_{1}$ or $A_{2}$ are especially different, the profile of the waveform approaches the sinusoidal wave. Sinusoidal vibration does not induce the illusion [9]. In this experiment, $A_{1}$ and $A_{2}$ were equivalent.

To design the fundamental frequency, we introduced a previous example that verified the relationship between the frequency of the asymmetric vibration and the illusion. Amemiya et al. evaluated the clarity of the illusion with two types of commercially available vibrators (Force Reactor, Alps Electric Co., Ltd., Tokyo, Japan and Haptuator, Tactile Labs Inc., Mont-Royal, QC, Canada) when the vibrators were fed with pulse width modulation (PWM) signals at 40 and 125 Hz [8]. It was shown that the combination of the Haptuator and the frequency of 40 Hz yielded the best outcomes. In addition, Culbertson et al. reported that this vibrator (Haptuator, Tactile Labs Inc.) can easily generate asymmetric vibrations at 40 Hz [6]. The authors evaluated the direction discrimination of the illusion with the vibrator (Vibro Transducer Vp210, Acouve Lab Inc.) when this vibrator was used to input a signal that inverted part of sine wave (frequencies: 50, 75, and 100 Hz) [9]. As a result, the frequency of 75 Hz yielded the best performance. In addition, in the basic model of the asymmetric vibration (Equation (1)), we confirmed the effects of the phase difference at 75 Hz [18]. Thus, the main frequencies at which the illusion occurred were 40 Hz [6,8] and 75 Hz [9,18]. In this experiment, the fundamental frequency *f* was 40 Hz and 75 Hz (2 levels) because the illusion occurred at these frequencies. If the nonlinear sensory properties of humans contribute to the pulling illusion, the illusion may occur when the phase difference is −180 deg and 0 deg, even at 40 Hz, similar to response generated at 75 Hz. Therefore, the phase differences $\phi_{0}$ were −180, −90, 0, and 90 deg (four levels). The amplitudes of the vibration acceleration $A_{1}$ and $A_{2}$ were set to 40 $m/s^{2}$, based on the results of the preliminary experiments.

#### 2.1.3. Apparatus

To isolate and clarify the effects of frequency alone, the asymmetry of the vibration waveform and frequency must be controlled independently. For that purpose, precise control of asymmetric vibration is required. To generate the asymmetric vibration stimuli, the voice-coil type vibrator developed by us was used to evaluate the illusion [18]. The frequency response of this device is flat in the tested frequency range so that the pulling illusion may occur, and so vibrations can be controlled based on the dynamics of the finger for each participant. By considering the mechanical characteristics of vibrator and fingers, it is possible to clarify the effect of frequency based on perceptual characteristics of a human that previous studies [8,9] could not clarify. More details of our device can be found in [18].

**Figure 3:**
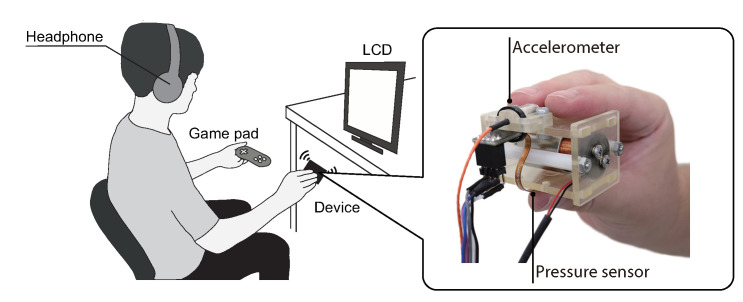
Experimental setup.

To measure the vibration acceleration of the device, an accelerometer (Type-4517, Brüel & Kjær), an amplifier (Type-2693-0S1, Brüel & Kjær), and a multifunctional data acquisition (DAQ) device (USB-6343, National Instruments Co., Austin, TX, USA) were used. The accelerometer was attached to the handle of the device. The sampling frequency of the DAQ device was 20 kHz. The measured acceleration data was smoothed with a three-order Butterworth low-pass filter with a cut-off frequency a 5 kHz. The device was pinched with the use of the thumb, index finger, and middle finger, as shown in Figure 3 because this illusion was induced by pinching the vibrator [9]. To measure the gripping force of the participant during the psychophysical experiment, a pressure sensor (SingleTact S8-10N, Pressure Profile Systems, Inc., LA, CA, USA) was attached between the thumb and the device. Since more details of the control method of asymmetric vibration can be found in [18], they are omitted.

#### 2.1.4. Procedure

Figure 3 shows the experimental setup. Participants sit on the chair and gripped the device with dominant hand. A gamepad (JC-U3808TWH, ELECOM Co., Ltd., Osaka, Japan) was held in the nondominant hand to indicate the perceived force direction with its cross-key. To maintain a constant gripping force, a liquid crystal display (LCD) displayed the target gripping force and current gripping force. Before the psychophysical experiment, a procedure to maintain the grip force constant and a procedure to calibrate the stimulus were performed. More details of these procedures can be found in [18]. Audio information was suppressed by a noise-canceling headset (WH-1000XM2, Sony Corp., Tokyo, Japan) that output white noise. During the experiment, the participants looked at an LCD screen that showed the gripping force.

A randomly selected stimulus was defined based on the selections from the two levels of the fundamental frequencies and the four levels of the phase differences. In this experiment, a two-alternative forced choice (2AFC) was used. Participants responded to the direction of the perceived force based on the terms “to the right” or “to the left.” The ratio of “to the right” answers for each condition was calculated. Each stimulus was presented for 1 s and the next vibration was presented after the participant responded the direction of force and after a 2 s break. In total, 240 trials were performed by each participant, and 30 trials were conducted at each condition. In consideration of the fatigue that arose during the experiment and the adaptation to the stimulus, all trials were divided into six blocks of 40 trials each, and the participants were allowed two-minute breaks between successive blocks. The duration of the experiment was less than 90 min.

#### 2.1.5. Data Analysis

One-sample *t*-test was performed for the ratio of the “to the right” (hereinafter referred to as the ratio) for a chance level of 50% (because the 2AFC was used). A two-way repeated-measures analysis of variance of the ratio (ANOVA, factors: fundamental frequency and phase difference) was performed. If the Mauchly’s sphericity test was significant, the Greenhouse–Geisser correction was applied. A multiple-comparison test (Bonferroni method) was performed as a post-hoc test.

### 2.2. Results

First, the accuracy of the stimulus is reported. Figure 2 shows a typical example of the acceleration profile of a participant, averaged over all trials. The red lines indicate the averaged acceleration of the measured data, the dotted lines indicate the target, and the envelope indicates the standard deviation. The phase differences were calculated from the phases of the fundamental wave and the second harmonic wave after the transformation of the time series data of the asymmetric vibration with the fast Fourier transform (FFT). The root-mean-squared error (RMSE) was calculated between the target and measured phase differences at each condition. As a result, the RMSE values were 7.37 deg at 40 Hz and 4.85 deg at 75 Hz.

**Figure 4:**
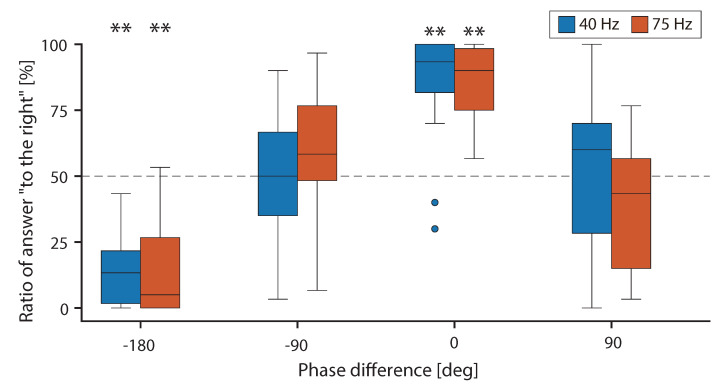
Plots of ratios of answers “to the right” at each condition.

Figure 4 shows the ratio at each condition. The top and bottom parts of the box indicate the lower and upper quartiles of the ratio, the whiskers indicate the minimum and maximum values of the ratio, and the horizontal bar indicates the median of the ratio. The dots indicate the ratios from the lower quartile −1.5× interquartile range (IQR) or the upper quartile +1.5×IQR. First, the results of the one-sample *t*-test are reported. The symbol “**” in Figure 4 indicates the conditions that yielded significant differences in chance level. The significant differences were found to be equal to −180 and 0 deg at 40 and 75 Hz (p<0.01). Therefore, the illusion occurred when the phase differences were -180 and 0 deg at both frequencies. As a result of ANOVA, the main effect of the phase difference [$F\left(2.14,32.14\right)=38.46,p<0.01,\eta_{p}^{2}=0.72$] and the interaction between the frequency and the phase difference [$F\left(3,45\right)=2.94,p<0.05,\eta_{p}^{2}=0.16$] were significant, and the main effect of the frequency was not significant [$F\left(1,15\right)=0.003,p=0.96,\eta_{p}^{2}=0.00$]. Subsequently, subeffect tests were performed because the interaction was significant. The simple main effect of the phase difference was significant at both 40 Hz [$F\left(3,13\right)=30.05,p<0.01,\eta_{p}^{2}=0.88$] and 75 Hz [$F\left(3,13\right)=22.53,p<0.01,\eta_{p}^{2}=0.84$]. As a result of the multiple comparison test for phase differences in each frequency, significant differences were observed in all tested combinations except −90 and 90 deg at 40 and 75 Hz (p<0.01). Although the interactions were significant, the combinations associated with significant differences were the same at both tested frequencies. In addition, the simple main effects of the frequency in each of the phase differences were not significant at −180 deg [$F\left(1,15\right)=0.11,p=0.75,\eta_{p}^{2}=0.007$], −90 deg [$F\left(1,15\right)=1.05,p=0.32,\eta_{p}^{2}=0.07$], 0 deg [$F\left(1,15\right)=0.05,p=0.82,\eta_{p}^{2}=0.003$], and 90 deg [$F\left(1,15\right)=2.27,p=0.15,\eta_{p}^{2}=0.13$]. Thus, it was suggested that the effects of frequency were small on the illusion and phase differences at different frequencies. Therefore, even at 40 Hz, it was shown that the difference in the change rate of acceleration for each polarity contributed to the illusion. As 40 Hz [6,8] and 75 Hz [9,18] were the main frequencies at which illusion occurred, it is expected that similar results will be observed at other frequencies where illusion may occur. This means that the relationship between illusion and frequency can be evaluated by changing the fundamental frequency *f* in the basic model of the asymmetric vibration waveform (Equation (1)) because the effect of frequency is small in the relationship between the illusion and the vibration waveform.

## 3. Experiment II: The Effect of Frequency Against Threshold that Induced the Pulling Illusion

Experiment II evaluated the relationship between the fundamental frequency and the illusion. From the results of Experiment I, it was confirmed that the illusion occurred with a high probability if the amplitude of the acceleration (intensity) of asymmetric vibration was large. It is difficult to clarify the effect of frequency based on simplified force discrimination as a function of the force direction. Therefore, in Experiment II, the effect of frequency against the pulling illusion was clarified based on the measurement of the threshold that induced the illusion. We aimed to demonstrate the changes of the threshold of the illusion at different frequencies. It was assumed that the participants could perceive the changes of the direction of the force when the illusion occurred. Thus, the threshold of the pulling illusion was defined as the minimum intensity of vibration at which participants could perceive changes in the direction of force.

### 3.1. Method

#### 3.1.1. Participants

Sixteen participants aged 21–56 years (mean: 30.1 years, SD: 12.8 years, two female) participated in the experiment, and all were right-handed. This experiment was approved by the institutional review board of the National Institute of Advanced Industrial Science and Technology (AIST), and the procedures were performed in accordance with the Declaration of Helsinki. Informed consent was obtained from all individual participants included in the study.

#### 3.1.2. Stimuli

In this experiment, the fundamental frequency *f* in Equation (1) was changed to clarify the relationship between the illusion and frequency. The frequencies of 40 and 75 Hz were used because the illusion will occur at these frequencies. In addition, a previous study discussed that the Pacinian corpuscles, the response to high frequency vibration, did not contribute to the illusion because they cannot sense the direction of vibration [6]. Based on this discussion, it is expected that the threshold of the illusion increases as the frequency of the asymmetric vibration increases. Therefore, to observe changes in the threshold of the illusion that corresponded to the fundamental frequency, the frequency of 110 Hz, that is the higher frequency of the same resolution between 40 Hz and 75 Hz, was also used. The condition of the fundamental frequency consisted of three levels, i.e., 40, 75, and 110 Hz (second harmonic: 80, 150, and 220 Hz).

**Figure 5:**
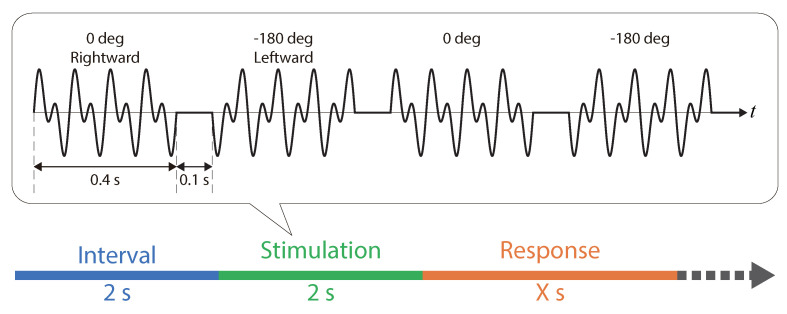
Stimulation and procedure.

Based on the threshold that we defined, stimuli were used such that the direction of the force repeatedly inverted on the left and right. In Equation (1), Experiment I confirmed that the illusion to the right occurred when the phase difference $\phi_{0}$ was 0 deg, while the illusion to the left occurred when $\phi_{0}$ was −180 deg. Thus, the direction of force reciprocated twice from the right (0 deg) to the left (−180 deg), or from the left to the right (Figure 5). Each direction was presented for 0.4 s. This period corresponded to the stimulation time period that could allow a sufficient force discrimination [9]. An interval of 0.1 s was set between directions. The total stimulation time was 2 s ((0.4 + 0.1) s × two directions × two round-trips) per stimulation (Figure 5).

To evaluate the occurrence probability of the illusion that corresponded to the vibration intensity based on the method of constant-stimulus, the amplitude of the vibration acceleration was changed. The amplitudes of the vibration acceleration, $A_{1}$ and $A_{2}$, was set to 8, 16, 24, 32, and 40 $m/s^{2}$ (five levels), based on the results of the preliminary experiments.

#### 3.1.3. Apparatus and Procedure

Experiment II was performed with the same apparatus and environment as those used in Experiment I (Figure 3). Additionally, as shown in Experiment I, input signals to our device were generated for each participant before the psychophysical experiments.

A randomly selected stimulus was presented to the participants. The stimulus was selected among the three levels of the fundamental frequency conditions and the five levels of the vibration intensity conditions. In accordance with the procedure of the method of constant-stimulus, participants answered “Yes/No” with the 2AFC to the question “Can you feel the pulling force that was repeated in the leftward and rightward directions?”. Participants answered “Yes” only if they were able to perceive the direction of the inverted force. They answered “No” if they did not perceive the force, although they could perceive the stimulation when different vibrations were repeated. The next stimulation was presented after the participant answered, and after the provided break that lasted 2 s (Figure 5). In total, 300 trials were performed by each participant with 20 trials performed at each condition. In consideration of fatigue that could have arisen during the experiment and the required adaptation to the stimulus, all trials were divided into 10 blocks that comprised of 30 trials each. The participants were allowed to break for 2 min between successive blocks. The duration of the experiment was less than 120 min.

#### 3.1.4. Data Analysis

To calculate the threshold required to induce the illusion at each fundamental frequency, the ratios associated with those who answered “Yes” (hereinafter referred to the occurrence probability) were calculated in each intensity condition, and a psychometric function was acquired based on the fitting of the occurrence probability to the cumulative normal distribution function with the least-squares method. The intensity at the 50% point of the psychometric function was used as the threshold. Considering the error between the target and measured values in the intensity of the asymmetric vibration stimulus, the measured value of the intensity was used when the psychometric functions were fitted. The intensities (amplitude of acceleration) were calculated after the transformation of the time series data of the asymmetric vibration with the FFT. Accordingly, the average value of each condition was used as the measured intensity. In addition, assuming that the occurrence probability was 0% when the amplitude of the acceleration was zero ($A_{1}=A_{2}=0$), this point was added to the previously collected five-point measurements, and the psychometric functions were fitted with the use of six points.

A one-way repeated-measures ANOVA of the threshold (factor: fundamental frequency) was performed. A multiple comparison test (Bonferroni method) was performed as a post-hoc test.

### 3.2. Results

**Figure 6:**
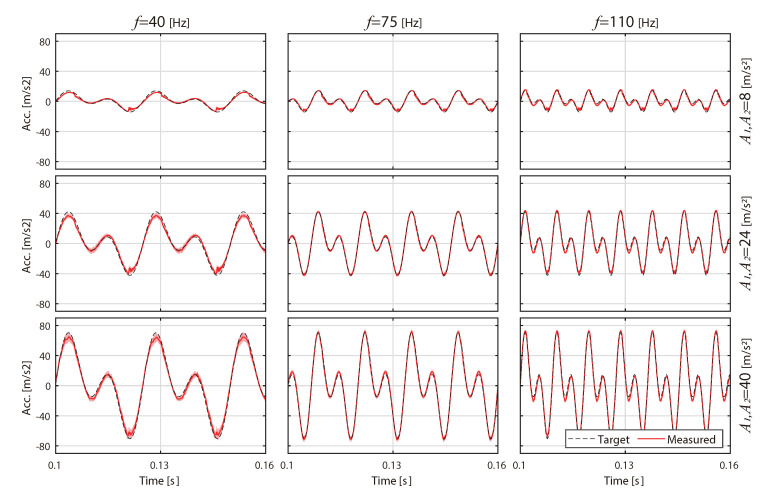
Typical example of an acceleration profile of the fingertips (in Equation (1), f=40, 75, 110 Hz, $\phi_{0}=$0 deg, $A_{1}=A_{2}=$8, 24, 40 $m/s^{2}$).

First, the accuracy of the stimulus is reported. Figure 6 shows a typical example of a participant’s acceleration profile averaged over all the conducted trials. The RMSE values between the target and measured phase differences were 8.94 deg at 40 Hz, 6.98 deg at 75 Hz, and 11.52 deg at 110 Hz.

**Figure 7:**
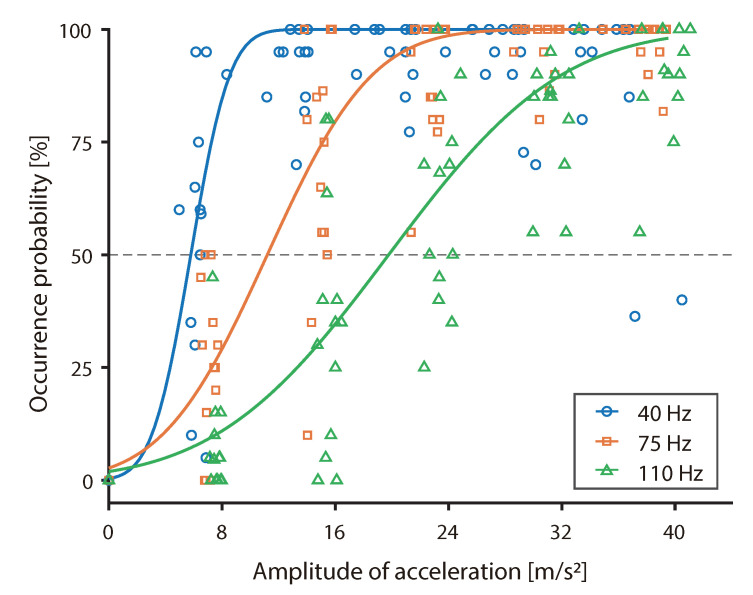
Scatter plot of the occurrence probability, amplitude of vibration acceleration, and typical examples of psychometric functions.

Figure 7 shows a scatter plot of the occurrence probability and amplitude of acceleration. The solid line on the graph shows a typical example of the psychometric function for each fundamental wave frequency that fitted the median value of thirteen participants. The occurrence probability of the three participants was less than 50% in all conditions. These three participants were excluded from the analysis because the psychometric functions could not be fitted. Two of the three observed a decreasing occurrence probability tendency as a function of the decreasing vibration intensity. The occurrence probability of another participant was 100% at all conditions. Therefore, it is considered that the thresholds associated with these three participants existed at points other than that associated with the vibration intensity setting.

**Figure 8:**
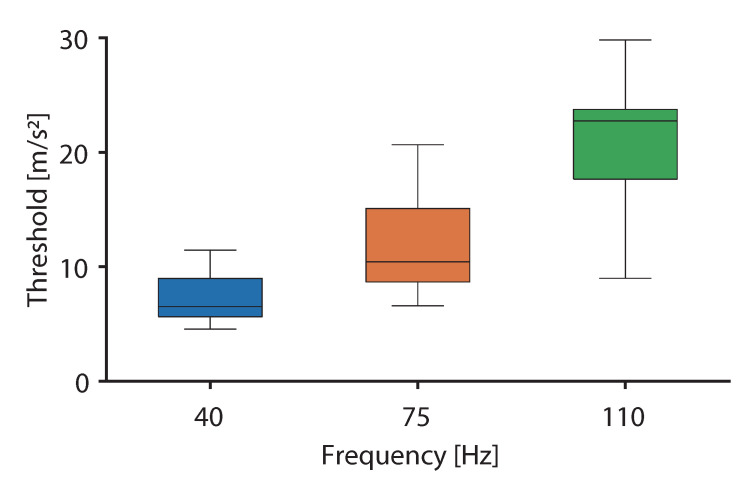
Thresholds of illusion occurrences at different frequencies.

Figure 8 shows the threshold based on which the illusion occurred at each fundamental frequency. The top and bottom parts of the box indicate the lower and upper quartiles, the whiskers indicate the minimum and maximum values, and the horizontal bar indicates the median of the occurrence probability. It was confirmed that the threshold increased as the fundamental frequency of asymmetric vibration increased. As a result of the ANOVA, the main effects of the fundamental frequency were significant [$F\left(2,24\right)=47.99,p<0.01,\eta_{p}^{2}=0.80$]. As a result of the multiple comparison test, significant differences were observed for all the tested combinations (p<0.01).

## 4. Discussion

### 4.1. General Discussion

In Experiment I, it was shown that the illusion was induced with the same vibration waveform given that the phase differences were −180 deg and 0 deg at 40 Hz and 75 Hz. Moreover, the illusion was induced when the fundamental frequency was 110 Hz with the use of the same vibration waveform in Experiment II. When the fundamental frequency of asymmetric vibration changed from 40 Hz to 110 Hz, the illusion was induced by asymmetric vibrations and the change rates of acceleration for each polarity were different.

From the results of Experiment I, the effect of the fundamental frequency was small against the pulling illusion when the vibration intensity was sufficiently large. However, the threshold at which the illusion occurred varied as a function of the fundamental frequency given the results of Experiment II. It is suggested that the threshold of the pulling illusion depends on the fundamental frequency of the asymmetric vibration. The reason is discussed below. The major mechanoreceptors are Merkel disks, Meissner corpuscles, Ruffini endings and Pacinian corpuscles [15]. Meissner corpuscles and Ruffini endings are sensitive to changes in the tangential direction [19]. Therefore, Amemiya et al. hypothesized that the Meissner corpuscles and the Ruffini endings contribute to the illusion [8,19]. They designed the asymmetric vibration so that the fundamental frequency was 40 Hz [8] given that this was the frequency at which these mechanoreceptors were likely to respond to [15]. Conversely, although it is known that the Pacinian corpuscles can sense the high frequency vibration (100–300 Hz) [15], these receptors cannot sense the direction of vibration [20]. Therefore, previous studies indicated that the Pacinian corpuscles do not contribute to the illusion [6,8]. As a result of Experiment II, the threshold was the lowest when the fundamental frequency was 40 Hz, and the threshold was the highest when the frequency was 110 Hz. This result supports the hypothesis that the Meissner corpuscles and the Ruffini endings contribute to the illusion, while the Pacinian corpuscles do not. Therefore, the characteristics of the mechanoreceptor may contribute to the illusion. We conclude that the illusion may be induced with a smaller amount of energy based on the use of frequencies in the band that spans several tens of Hz, wherein both the Meissner corpuscles and Rufini endings are likely to respond. By contrast, because the illusion can occur even at 110 Hz, this is not necessary as long as sufficient vibrations can be presented.

The findings on the relationship between the fundamental frequency and the pulling illusion constitute the guidelines for the design of the frequency band of the actuator for the development of haptic interfaces. However, the frequency range within which the illusion occurs, and the frequency at which the threshold is minimized have not been clarified. In the future, an evaluation study of the illusion in a wider frequency band will be conducted. Although the measurement of the threshold at which the illusion occurred is effective in the evaluation of the effects of the fundamental frequency, the measurements conducted with the method of constant-stimulus required time. Experiment II measured thresholds only at three frequencies owing to the time constraints of the experiment. To evaluate the illusion in a wider frequency band, another threshold measurement method may be useful.

In this study, the acceleration of vibration was controlled based on the concept from Amemiya el al. according to which the illusion was induced by asymmetric acceleration [1], and the acceleration was used as a unit of vibration energy [21]. By contrast, in terms of the vibration displacement, 40 Hz yielded the maximum displacement and 110 Hz yielded the minimum displacement, as the acceleration was the same at the three frequencies. In addition to the hypothesis that the threshold changed owing to the characteristics of the mechanoreceptors, it is likely that the threshold in the vibration acceleration level was the lowest because the displacement reached its maximum at 40 Hz. Moreover, the vibration stimulus of the fingertip propagated to regions near the base of the finger at lower frequencies than at higher frequencies [22]. The threshold may have been minimized by the propagation of vibration near the base of the finger at 40 Hz. Therefore, further investigation with controlled displacements and measured vibration at multiple points is required.

### 4.2. Discussion of Individual Differences

**Figure 9:**
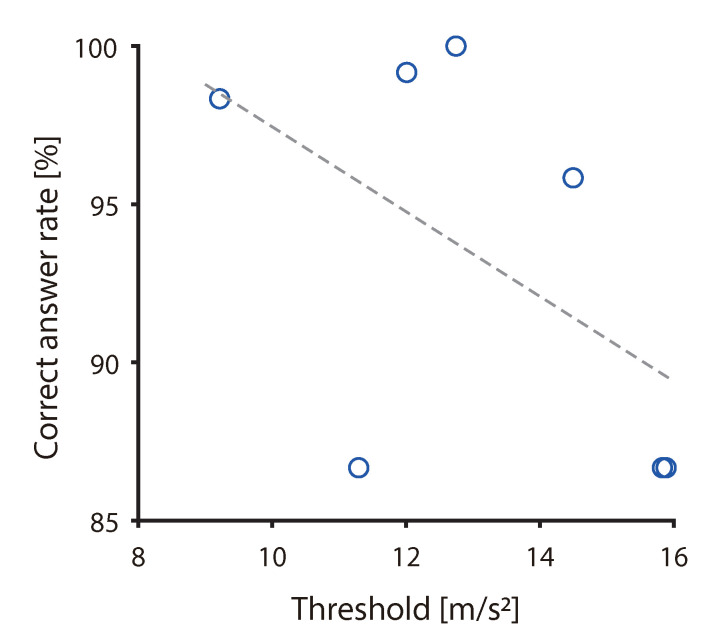
Correlation between threshold and correct answer rate.

Seven of all participants participated in both Experiments I and II. Among these seven participants, the correlations of all experimental results were analyzed (Figure 9). In the results generated in Experiment I, the ratio at −180 and 0 deg was considered as the correct answer rate. In the results generated in Experiment II, the average of the thresholds at all frequencies was used. As a result, a negative correlation was observed (r=−0.52,p=0.22), even if the sample size was small. Therefore, it was suggested that participants with a low threshold were able to determine the direction of the pulling force, and the correct answer rate was thus high. One of the participants excluded from the analysis in Experiment II had a 100% occurrence probability at all conditions. This participant indicated a piano playing experience that spanned 20 years. Accordingly, the participant’s sensitivity to tactile sensation may have been higher than others. When playing the piano, not only auditory but also tactile information from the fingertips is important. As a result that this participant used tactile sensation more frequently than others, the illusion might have been induced with a probability of 100%. Therefore, individual differences in tactile sensation inherent in each person may affect the threshold of illusion, and it may also affect the discrimination direction of the force. By evaluating the illusion and by measuring the vibrotactile threshold that allowed perception of the low-frequency vibration (that corresponded to the Meissner corpuscles and the Ruffini endings) or high-frequency vibrations (that corresponded to the Pacinian corpuscles) at the same time, it may be possible to clarify the factors responsible for individual differences and the mechanoreceptors that contributed to the illusion.

## 5. Conclusions

In this study, we clarified the effects on the pulling illusion from the difference in frequency of the asymmetric vibration. As a result of Experiment I, it was shown that the illusion occurred at specific vibration waveforms at 40 Hz and 75 Hz. As a result of Experiment II, it was shown that the threshold at which the illusion occurred increased as the fundamental frequency of asymmetric vibration increased. It was suggested that the Meissner corpuscles and the Ruffini endings contributed to the illusion, while the Pacinian corpuscles did not. Therefore, to generate the illusion, the latter may have been induced with a smaller amount of energy with the use of frequencies in a band that spanned several tens of Hz. These findings are expected to assist the designs of haptic interfaces that are based on the concept of pulling illusion. However, the frequency range within which the illusion occurred and the frequency at which the threshold was minimized have not been clarified. In the future, we will evaluate the illusion within a wider frequency band.
